# Histological response of soda-lime glass-ceramic bactericidal rods implanted in the jaws of beagle dogs

**DOI:** 10.1038/srep31478

**Published:** 2016-08-12

**Authors:** José S. Moya, Arturo Martínez, Roberto López-Píriz, Francisco Guitián, Luis A. Díaz, Leticia Esteban-Tejeda, Belén Cabal, Federico Sket, Elisa Fernández-García, Antoni P. Tomsia, Ramón Torrecillas

**Affiliations:** 1 Nanomaterials and Nanotechnology Research Center (CINN), CSIC - University of Oviedo (UO), Avda de la Vega 4-6, El Entrego 33940, San Martín del Rey Aurelio, Spain; 2 Institute of Materials Science of Madrid (ICMM-CSIC), Cantoblanco, 28049, Madrid, Spain; 3 Galician Institute of Ceramics, University of Santiago de Compostela, Avda Maestro Mateo, 15782 Santiago de Compostela, Spain; 4 IMDEA Materials Institute, C/ Eric Kandel 2, Getafe, 28906, Madrid, Spain; 5 Materials Sciences Division, Lawrence Berkeley National Laboratory, Berkeley, CA 94720, USA

## Abstract

Bacterial and fungal infections remain a major clinical challenge. Implant infections very often require complicated revision procedures that are troublesome to patients and costly to the healthcare system. Innovative approaches to tackle infections are urgently needed. We investigated the histological response of novel free P_2_O_5_ glass-ceramic rods implanted in the jaws of beagle dogs. Due to the particular percolated morphology of this glass-ceramic, the dissolution of the rods in the animal body environment and the immature bone formation during the fourth months of implantation maintained the integrity of the glass-ceramic rod. No clinical signs of inflammation took place in any of the beagle dogs during the four months of implantation. This new glass-ceramic biomaterial with inherent bactericidal and fungicidal properties can be considered as an appealing candidate for bone tissue engineering.

The risk of bacterial and fungal infections from medical devices inserted or implanted in the body has been well documented in literature^1^,^2^,^3^,^4^ and remains a critical unsolved issue that has significant clinical and economic consequences. In the United States alone, 112,000 out of 2,600,000 orthopedic implants inserted annually have resulted in infections with an estimated average cost of combined medical and surgical treatments coming to $45,000 per device^1^.

In spite of various preventive methods, such as antibiotic prophylaxis and the use of gloves, drapes, masks, and ultraviolet light during surgery and post-surgical wound care, the implants themselves are still susceptible to developing infections near their surface. This is due to the formation of a biofilm on the implant surface that induces a patient’s immune response at the implant/tissue interface. In addition, bacteria in biofilms are protected from the immune system, and they are usually resistant to antibiotics^3^,^5^,^6^. Thus, one of the most critical problems in the current healthcare system is the prevention of bacterial adhesion to implant surfaces.

Recently, the main area of research into inorganic biocides concerns itself with the fields of nanoscience and nanotechnology, in which the approach is mainly directed toward developing materials containing silver or copper nanoparticles^7^,^8^,^9^. Lately, bioactive glasses are also being investigated as novel carrier systems for treating implant infections. One approach to conferring bioactive glasses with antimicrobial activity is by doping them with antibacterial agents, such as silver, copper, and titanium; or by loading them with antibiotics. These elements are incorporated into the glass during the manufacturing process, and as the glass degrades *in vivo*, ions of those elements are released at a clinically desirable rate^10^. However, there is a negative impact of metal-based nanomaterials: metal and metal-oxide nanoparticles may be toxic to humans^11^, and the extent of such toxic health effects is still unknown^12^,^13^. In addition, the diverse array of metal and metal oxides, the different sizes and shapes that can be prepared from metal-based nanomaterials, and the variety of possible surface coatings made from these materials make it even more difficult to assess toxicity^14^.

One way to reduce the risks of toxicity is to exploit a new generation of inorganic biocides based on soda-lime glass powders^15^ with a composition similar to that of conventional window glass. These glass powders, enriched with CaO, have been converted into a high-added-value material suitable for application in several strategic fields in which the control of an unwanted heterogeneous microbial population represents a serious threat to human health. The results obtained have been unexpectedly positive: The biocidal activities of these glass powders have been exceptionally effective when evaluated against Gram-positive bacteria (*Micrococcus luteus*), Gram-negative bacteria (*Escherichia coli JM 110*), and yeast (*Candida krusei*). The efficacy of a coating made of this kind of glass to prevent peri-implant diseases was pointed out in a previous *in vivo* study^16^, in which different bactericidal glassy coatings on zirconia abutments were connected to dental implants inserted on the jaws of beagle dogs. This glassy coating was able to prevent biofilm formation, to inhibit intrasulcular bacterial growth and to prevent peri-implant marginal crestal bone loss. This glassy coating was effective preventing anaerobic bacterial growth, which are the ones directly associated with the peri-implant disease. The extremely high biocidal activity of these glass powders is very encouraging and opens the door for their use in several specific medical applications. They may also serve as an alternative to other more aggressive inorganic compounds, such as silver and copper nanoparticles, which are still being considered.

In our previous works^17^,^18^,^19^ a new glass-ceramic composed of combeite and nepheline in a residual soda-lime glassy matrix was fabricated by sintering at 750 °C for 1 hour. The antibacterial activity of these glass-ceramics, free of P_2_O_5_, clearly shows that this new family of biomaterials effectively diminishes the growth (logarithm of reduction >3) of bacteria (such as *S. aureus*, *E. coli*, *S. epidermidis*, *M. lutea*, and *P. aeruginosa*)^17^ as well as yeast (*C. krusei*)^19^. Yeast in particular is quite dangerous: it causes severe problems in hospitals due to its growth in catheters and resistance to multiple antibiotics, and the well-established 45S5 Bioglass^®^ has almost no activity against yeast. In contrast, our new glass-ceramic exhibits a high biocide activity against *C. krusei* (logarithm of reduction >5)^19^. Furthermore, it has also been shown that this glass-ceramic inhibits adhesion and biofilm formation, a critical issue in several medical applications^17^. Comparison with the effectiveness other commercial materials to inhibit biofilm formation is not available yet, it is part of a work that is ongoing. On the other hand, the *in vitro* biocompatibility of assays with human stem cells demonstrates that the new glass-ceramic has an excellent biocompatibility^17^.

The present investigation is focused on the histological study of this new glass-ceramic in the form of a series of rods, implanted in the jaws of beagle dogs for a four-month period.

## Results

**Figure 1:**
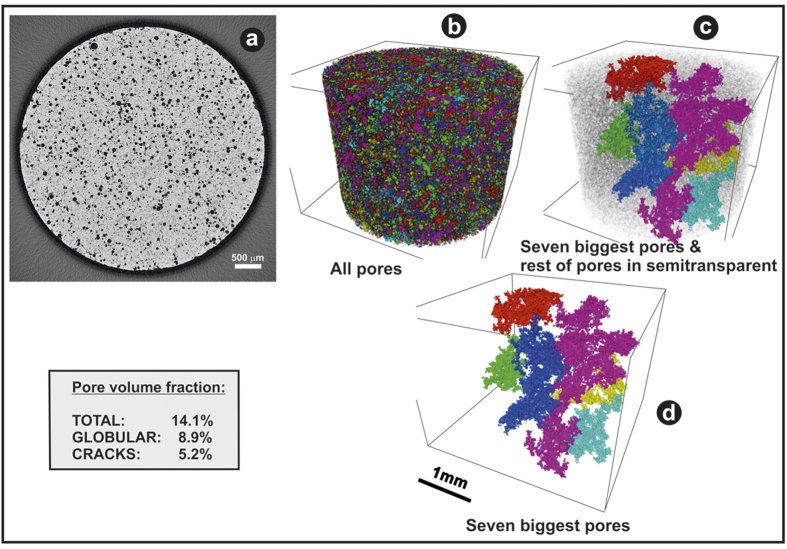
X-ray computed tomography (XCT) images of the cross section of the implanted rod (**a**). Volume reconstruction of all the pores in the sample (colours indicates different pores) (**b**). The seven biggest pores in the sample with the rest of the pores in semitransparent for dimension comparison (**c**). Only the seven biggest pores in the sample (**d**). In Figure (**b**–**d**) the material was set to transparent.

The X-ray computed tomography (XCT) 3D study of the as-sintered implant rods (Fig. 1) shows that globular pores (Vg = 8.9 vol%) are interconnected by micro-cracks (Vc = 5.2 vol%), and that the total porosity is 14.1 vol%. Globular pores and microcracks were separated by image analysis with a segmentation algorithm based on morphological operations. An example of the long range interconnectivity can be observed in Fig. 1d where the seven biggest pores (considering globular and microcraks pores) in the analysed volume are shown. It is worth mentioning that the fifty largest pores in the volume concentrate 20% of the total porosity and the total number of pores was ~2.2e6 pores. The average flexural strength of these rods was found to be σ = 40 ± 7 MPa.

**Figure 2:**
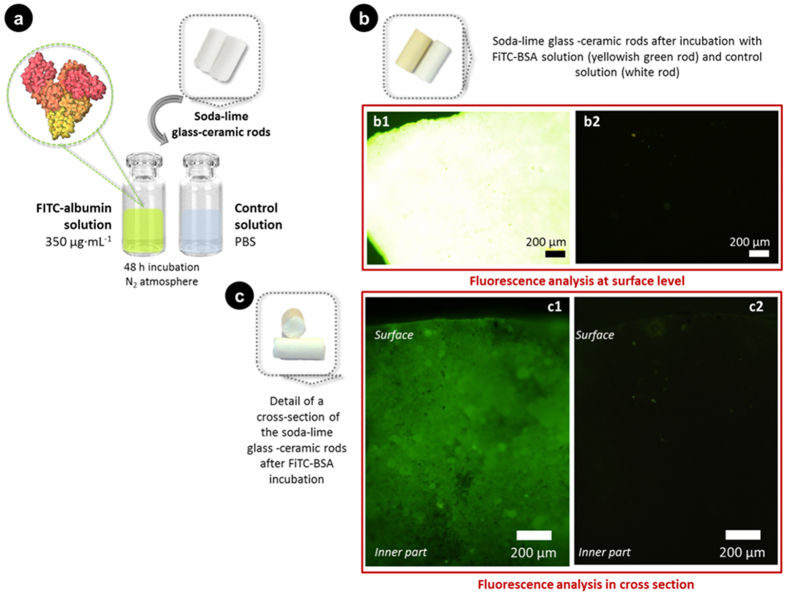
Permeability of a fluorescently labelled BSA throughout the porous glass-ceramic rod. A testing diagram (**a**) and images of the rod surface (**b**) and of the cross-section (**c**) after the test are shown. Green-fluorescence images were obtained from the rod surface (**b1**) and in cross section (**c1**) after incubation with FITC-albumin for 48 h; control rods (**b2**,**c2**) were immersed in PBS with the same test conditions. The appearance of the rods at each state is also included in (**a**–**c**).

The interconnected porosity (Fig. 1) could allow the biological fluids flow through the implant structure^20^,^21^,^22^ (see Supplementary Figure 1). Additional *in vitro* tests were carried out in order to evaluate the possible diffusion of proteins toward the inner part of rods. Figure 2 shows a diagram depicting the test conditions (Fig. 2a), and the fluorescence images acquired after immersion in the FITC-albumin solution. They revealed the green probe on rod surfaces (Fig. 2b1) and on the cross-sections (Fig. 2c1). A detail of the rods before and after incubation with the green-labeled protein is also presented in (Fig. 2a,b), respectively. A high adsorption of proteins at the rod surface is revealed by a yellowish-green color after incubation of rods with FITC-albumin (Fig. 2b). This led to fluorescence saturation when the surfaces were analyzed by fluorescence microscopy (Fig. 2b1). In addition, Fig. 2c1 shows a representative fluorescence image acquired on a cross section, which is compared to that of control rods immersed in buffer solution. The green-fluorescence displayed along the fracture surface (Fig. 2c1) may only be explained as a result of a protein flow toward the inner parts throughout the interconnected network of pores that exists in this material. This suggests that physiological fluids may get absorbed at the rod surface, may penetrate through the cracks/pores, and also would be able to spread internally all over the pore network. As expected, no fluorescence was disclosed in those samples used as reference (analysis of rod surface and cross section in Fig. 2b2,c2, respectively). On the other hand, the passive infiltration of proteins inside the rod was also evaluated by immersion of specimens in cell culture medium containing 10% new born calf serum, for 48 h. The amount of proteins extracted from the rod after immersion in 10% serum-containing DMEM solutions was 4.175 ± 0.057 *μ*g·mm^−3^. This concentration was statistically higher (p ≤ 0.05) than that measured in reference rods (1.447 ± 0.011 *μ*g·mm^−3^). A quantity of proteins almost 3 times higher in the porous rod may be attributed to the filtration of supplemented DMEM medium to the inner part using the porous network. This finding would fit with the results in the previous experiment, i.e. the green-fluorescent labeling inside the rod that confirms an interior diffusion of fluids.

**Figure 3:**
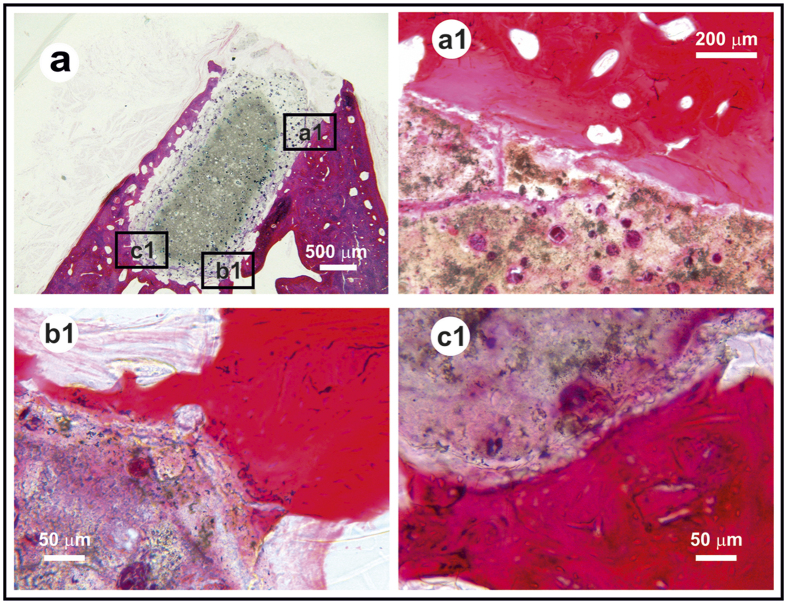
Histological image of the glass-ceramic rod (**a**). Optical micrographs showing interconnected microporosity allowing the migration of plasma fluid through capilar effect (**a1**) and details of the new bone-implant interface (**b1,c1**).

**Figure 4:**
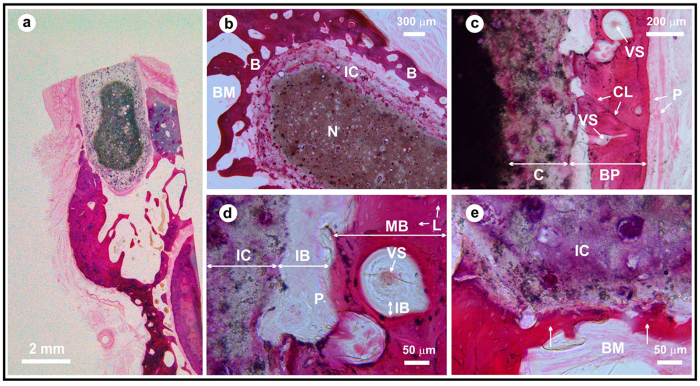
Histological images of the glass-ceramic rod showing bone osseointegration (**a**). Optical micrographs: Apical area of the implant (**b**), detail of the buccal plate of the alveolar bone on the surface of the implant (**c**), detail of the bone-implant interface (**d**) and detail of the interface where one can appreciate the appearance/growth of a bridge of mineralized bone tissue on the implant surface. Pores filled with calcium and organic compounds are observable within the crust (arrows) (**e**). Abbreviations: -N- core material, -B- new bone, -BM- bone marrow, -IC- implant, -BP- buccal plate of the alveolar bone, -VS- vascular areas of the neo-formed bone, -CL- cemental bone remodeling line, -C- partially dissolved surface layer of the rod, -P- periosteum, -IB- immature bone, -L- osteocytarieslacunaes, -MB- immature mineralized bone.

Bone cells precursors interact with the implant surface to lead the new bone formation. This was evaluated by an exhaustive histological assessment (Figs 3 and 4). Figure 3 is focus on the analysis of bone/implant interface and Fig. 4 discloses the new bone tissue formation. Certain cracking (Fig. 3a,1) is observed at that level likely due to forces involved in chewing. The histomorphometric study (Fig. 3b,1,c1) gives rise that the fraction with direct apposition to the bone is: 35.0 ± 3.5%. After four months, the average amount of new immature bone plus the apparent dissolved/attacked rod layer in the implanted rods, was found to be 70 ± 14 vol% of the total volume of the rod (Fig. 4). This value was determined by a simple geometrical calculation by the difference between the initial implant volume (3 mm in diameter and 6 mm in length) and the undissolved/pristine (core) volume fraction of the rod. In order to estimate the amount of immature bone formation with our glass-ceramic implant versus commercial products such as deproteinized bovine bone (DBB) and tricalcium phosphate (TCP) scaffolds additional experiments are required and this will be the subject of a future work.

The main results obtained from the histological analysis can be summarized as follows: in the low magnification micrograph (Fig. 4a), a rectangular structure (approximately 2 × 3.5 mm) implanted in the alveolar ridge of the jaw was observed. The implant was positioned in the alveolar crest, under the buccal plate. No fibrous reaction at the implant-bone interface was observed, and bone–both cortical and cancellous– was closely positioned on the implant surface. Two parts are visible on the implant (Figs 3 and 4): a) a dense nucleus with spherical pores occupied by very dense, irregular structure aggregates, and b) an irregular crust with 100–400 μm in thickness having a lower density than the core.

**Figure 5:**
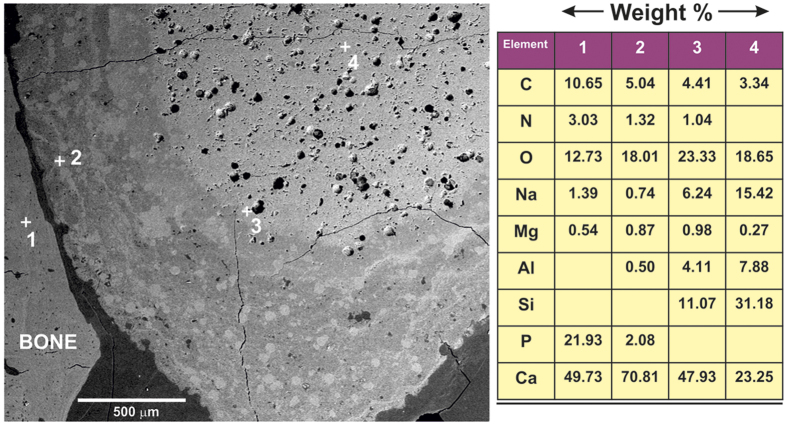
Field-emission scanning electron micrograph (FE-SEM) of the implanted rod and energy-dispersive X-ray spectroscopy (EDS) microanalysis of the selected spots, corresponding to numbers 1, 2, 3, 4.

**Figure 6:**
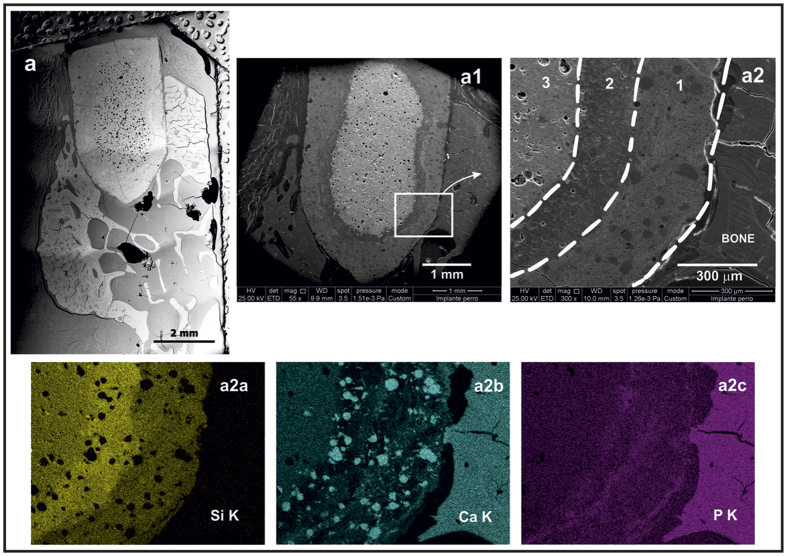
Backscattered field emission scanning electron microscopy (FESEM) image (**a**) showing a general view of the implant in contact with the bone at two magnifications (**a1**,**a2**). Energy-dispersive X-ray spectroscopy (EDS) maps of Si, Ca, and P (**a2a–a2c**).

The crust is subdivided into two different layers (Figs 5 and 6). The glass-ceramic rod or at least its glassy phase, in contact with the body fluids, is dissolved following an onion layer structure, basically through an ion-exchange process. The physiological environment extracts Na, K, and Ca ions, giving rise to an amorphous hydrated silica bands. The observed porous zone is formed from preexisting pores in the glass ceramic rod, their edges widening by dissolution. EDS analysis (Fig. 5) shows the existence of Ca, C, O and N in the globular pores, which indicate the presence of organic compounds. The rate of C and N decreased as we approach to the non-attack core of the glass-ceramic rod where the pristine composition still remains. Very high calcium content appears within the pores (Figs 6a and 2b). These results reveal the existence of gradient in the composition from the interface with immature bone to the pristine core of the rod.

## Discussion

The cracks-pores system observed in Fig. 1 is clearly percolated, thus permitting the body fluid to diffuse through the volume of the implant (Fig. 2 and Supplementary Figure 1) and furthermore reaching the globular pores (Fig. 2), facilitating the new bone formation in a controlled process. This particular microstructure of P_2_O_5_-free glass-ceramic is a consequence of the crystallization of both nepheline and combeite. The specific volume of these phases is different to the glassy matrix, which induces microcracking during the crystallization process.

This singular morphology has an important effect on the mechanical properties of the starting rods. For instance, in the case of the well-known 45S5 Bioglass^®^ sintered at >1000 °C, only combeite is present and the obtained mechanical properties were significantly lower (~50%)^18^,^19^, probably due to the absence of acicular nepheline crystals in the microstructure^23^.

The implanted glass was affected by dissolution/degradation processes. Previous work^24^ has reported that after soaking the glass in simulated body fluid (SBF), a surface layer of hydroxyapatite (HA) followed by an intermediate SiO_2_-rich layer was formed. Almost all pores were filled with calcium deposits (Figs 6a and 2b), which encourage bone growth^25^. A similar situation was described by L. Devesa *et al*.^26^.

Direct growth and bone formation at the implant surface was observed in the higher magnification micrographs (Figs 3 and 4d,e). There is a direct apposition of bone on the implant surface except in the gingival portion of the implant, as it is expected (Fig. 3). For the most part newly formed bone has grown on the implant surface, and occasionally it is separated from the old bone by a cement line (Fig. 4c). This new bone is less mineralized even so the osteocyte lacunae are distributed in a habitual fashion. The newly formed bone is undergoing normal remodeling processes with abundant vascular buds and basic multicellular units (BMU). Mainly, the newly formed bone is laminar and woven, and is not haversian (Fig. 4d,e). The bone marrow in contact with the implant is normal, and no vascular lesions were observed.

The occlusal surface of the implant was placed under periosteum and gingiva, giving rise to the formation of a dense layer of connective tissue that attaches at the material surface (Fig. 3a). Evidence of processes of foreign body response was not found nearby. Instead, there was new tissue that seals cracks (Fig. 3a,1). Specific attention is paid to the histological appearance at the rod/bone interface (Fig. 3b,1,c,1). A thick layer of fibrous tissue randomly organized that would suggest the encapsulation of this material was not observed; rather a solid union appeared all around. In overall, there was no signal of multinucleated giant cells or focal accumulation of cells that would indicate the presence of an immune reaction, suggesting a lack of immunogenicity. No atypia or dysplasia was observed.

**Figure 7:**
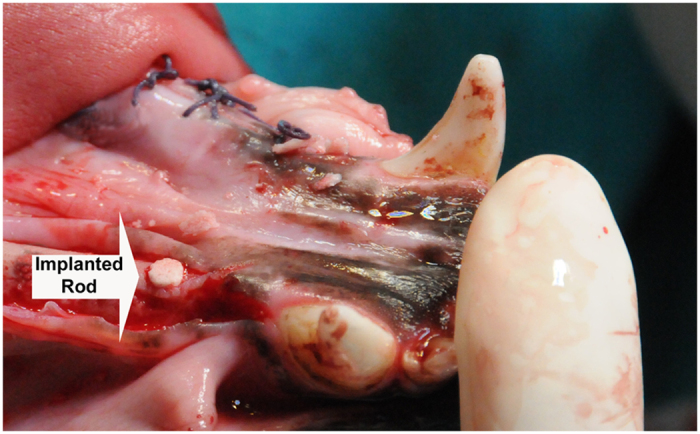
Image of a glass-ceramic rod, free of P_2_O_5_ implanted by impaction into the jaw of a beagle dog (photo taken by Roberto López-Píriz during the surgery).

Finally, it is important to state that during the four-month rod implantation in the beagles’jaws, even though the occlusal portion of the implant was exposed to the oral environment (Fig. 7) neither inflammatory clinical signs (redness, swelling or bleeding) were observed in any of the implants during our continuous clinical ocular inspections of the implants. These ocular inspections were performed daily during the 21 days due to the usual gingival swelling that take place in this period when infection occurs. After that the inspections were one by week. This absence of inflammation is related to the inherent antibacterial properties exhibited by the new free P_2_O_5_ free glass-ceramic^16^,^17^,^19^.

It is generally accepted that the degradation rate of bioglass ceramic implants/scaffolds must be compatible with new tissue in-growth, and that maturation as a consequence of long-term preclinical animal evaluation is crucial^27^. Degradation of bioactive glasses *in vivo* can be controlled to span weeks, months, or years, depending on their compositions^28^,^29^, while the HA ceramic scaffold still remains in the human body even seven years^30^. This behavior in conventional P_2_O_5_-containing bioglasses is due to the release of soluble Si, Ca, P, and Na ions as a result of glass-surface reaction and degradation in body fluids to give rise to the osteoinductive and osteogenic properties in bioactive glass^31^,^32^. Furthermore, bone formation can be enhanced in these glasses by doping with Sr due to its faster degradation rate and the release of the Sr ion, which is known to stimulate osteoblast activity while at the same time inhibiting osteoclast activity^31^.

Because of the absence of P_2_O_5_ and the presence of a small fraction of Al_2_O_3_ in our glass-ceramic during annealing at 750 °C for 1 hour, both crystal phases (combeite and nepheline) precipitated while the composition of the remaining continuous glassy phase remained similar to the one of the starting glass, with the same biocidal character^15^,^16^. In the case of the Hench series of glasses, only combeite is formed, and the remaining glassy phase has a completely different composition to the starting bioglass^33^.

Our alternative new P_2_O_5_ free glass-ceramic has the following advantages: better mechanical properties (σ = 40 ± 7 MPa) due to the crystallization of nepheline, a large spectrum of biocidal activity to prevent infections and biofilm formation^16^,^17^, an excellent balance between the degradation rate and new tissue in-growth.

## Conclusions

The following conclusions can be drawn: a) The P_2_O_5_-free glass-ceramic rods implanted in the jaws of beagle dogs due to their particular percolated morphology are subjected to a controlled dissolution/new-bone-formation process throughout the entire osseointegration process; b) as a direct consequence of the biocompatibility and the inherent bactericidal character of the glass-ceramics, no inflammatory process was observed in any of the beagles’jaws during our continuous ocular clinical inspections of the implants throughout the four-month rod implantation process.

## Methods

### Powder processing, mechanical and microstructural characterization

An antimicrobial soda-lime glass powder (d < 35 μm) from the SiO_2_–Na_2_O–Al_2_O_3_–CaO–B_2_O_3_ system with the following chemical composition was used (wt.%): 41.6 SiO_2_, 20.0 Na_2_O, 19.5 CaO, 10.1 Al_2_O_3_, 6.4 B_2_O_3_, 0.21 MgO, and 0.61 K_2_O (Nanoker Research, Spain).

The precursor powders were cold isostatically pressed (CIP) at 300 MPa as cylinders about 10 mm in diameter, and subsequently sintered in a conventional furnace in air at 750 °C for 1 hour. A mineralogical characterization of the sintered samples was made by X-ray diffraction (XRD, Bruker AXS D8 ADVANCE, with a SolX energy-dispersive detector) using CuKαradiation. The microstructure of sintered specimens after polishing down to 1 μm and machining was studied by scanning electron microscopy (FE-SEM, FEI Nova NANOSEM 230). The average bending strength of the machined rods was determined by a three-point bending test using 6 rods, 3 ± 0.2 mm in diameter and 15 mm long. The tests were performed at room temperature using a 5 kN universal testing machine, the Shimadzu AutoGraph AG-X (Kyoto, Japan), at a crosshead speed of 1 mm/min with a 12 mm span.

Three-dimensional non-destructive imaging of the implant rods was performed using a high-resolution X-ray CT system (GE Phoenix X-ray Systems, Nanotom 160 NF, GE, Germany). X-rays were produced with a 160 kV high-power nano-focus tube and transmission target configuration. A sample of ~4 mm in diameter was scanned. The projections were recorded in a 2300 × 2300 pixel Hamamatsu detector. The voltage and current in the tube were set to 90 kV and 135 μA, respectively, and X-rays were produced on a tungsten target. The voxel size used was (1.88 μm)^3^. We took 1,900 projections for the tomogram with a typical exposure time of 500 ms per projection. Each projection was obtained as an average of 10 radiographies. The total scanning time was about 3 hours. The X-ray tube of the “nanotom” is equipped with an external liquid cooling system to ensure stable measurement conditions and to minimize thermal influences during scans of long duration. The cone beam XCT data were reconstructed using a filtered back-projection Feldkamp algorithm implemented in the phoenix datos|x reconstruction software from GE Sensing & Inspection Technologies GmbH. A beam hardening correction was applied to decrease the strong dependence of the attenuation coefficient of the interacting material on the photon energy, thus the cupping artifacts were reduced. The reconstructed data were visualized with the VGStudio Max 2.2 software, and processed with ImageJ and in-house algorithms developed in Matlab.

### *In vivo* tests

#### Animals

This study was carried out in strict accordance with the Directive 2010/63/EU on the protection of animals used for scientific purposes. The protocol was approved by the Ethics Committee for Animal Research Welfare of the Minimally Invasive Surgery Centre (Cáceres - Spain) with Permit Number: 031/12. Five one-year-old beagle dogs were used in this experiment to accomplish with the goal of reduction, the second of the 3R’s (Replacement, Reduction and Refinement) widely accepted ethical framework for conducting scientific experiments. In addition, it is common practice to employ this number of animals for such experiments and thus satisfy statistical power requirements. Dogs at the initiation of the experiment were 12 months old and 13.5 kg mean weight.

Refining dog husbandry and care was provided throughout the study. During all procedures, veterinary assistance was used continuously and all efforts were made to minimize suffering. General anesthesia was induced with intravenous injected propofol 10 mg/kg (Propofol Hospira, Hospira Productos Farmacéuticos y Hospitalarios, Madrid, Spain). One no. 7 endotracheal tube with a balloon cuff was placed and connected to a circular anesthesia circuit (Leon Plus, Heinen&Löwenstein, Bad Ems, Germany). The anesthesia was sustained with sevofluorane (Sevorane, Abbott Laboratories, Madrid, Spain). Multimodal analgesia was employed in the perioperatory (ketorolac 1 mg/kg [Toradol 30 mg, Roche], tramadol 1.7 mg/kg [Adolonta injectable, Grünenthal], and buprenorfine 0.01 mg/kg [Buprex, Reckitt Benckiser Pharmaceuticals Limited, Berkshire, UK]).

#### Surgery

All mandibular premolars and the first molar were extracted from the five male beagles. After three months of healing, mucoperiosteal flaps were elevated, and 10 rods (3 ± 0.2 mm in diameter and 6 mm long) machined from the sintered cylinders were implanted by the rod impaction method. The rods were installed in the edentulous region on both sides of the mandible (see Fig. 7).

#### Histological Preparation and Analysis

After four months of implantation, the animals were euthanized with a lethal dose of sodium-penthotal. Mandibular blocks containing fixtures were retrieved and stored in a 5% formaldehyde solution (pH 7). The implant blocks were retrieved from the jawbone using an oscillating autopsy saw (Exakt, Kulzer, Germany). The dissected specimens were immediately immersed in a solution of 4% formaldehyde and 1% calcium and processed for ground sectioning following the Donath &Breuner methods^34^. Each implant block was labeled, embedded in methyl-methacrylate, and stained with a combination of Harris hematoxylin and Wheatley. The histological analysis was performed by using a transmitted light microscope (Optiphot 2-POL, Nikon, Japan) equipped with a digital camera (DP-12, Olympus, Japan).

### *In vitro* tests

#### Diffusion of green fluorescently labelled proteins through the porous rod

Sterilized rods (n = 3) were immersed in phosphate-buffered saline (PBS; 10 mM, pH 7.4) containing FITC-albumin (Albumin–fluorescein isothiocyanate conjugate, Sigma-Aldrich) as model protein. Serum albumin acts maintaining the oncotic pressure, it is a carrier for steroids, fatty acids, thyroid hormones, and many drugs; and it also binds competitively calcium ions and buffers pH. This serum protein is selected because of being the most abundant protein in human plasma. Bovine serum albumin presents a molecular weight of 66.5 kDa, and a diffusion coefficient of 1.86 ± 0.41 × 10^−7^ cm^2^·s^−1^ in fibrin scaffolds^35^. FITC-albumin is dissolved at 350 μg·ml^−1^ in order to simulate the blood albumin concentration, and is incubated with the rods for 48 h within nitrogen atmosphere. The specimens were rinsed after incubation and dried under nitrogen stream.

The serum albumin diffusion inside the rod is monitored by visualizing the green-fluorescent probe using microscope fluorescence (BHT system microscope, Olympus). FITC has a maximum absorption at 490 nm and an emission peak at 525 nm. The exposure times were set to 1.8 s in order to qualitatively evaluate the intensity differences. Fluorescence images were acquired on the material surfaces and on cross-section by analyzing fracture surfaces at 4x magnification. A parallel set of rods was immersed in PBS solution to be used as negative control.

#### Protein concentration

The passive infiltration of proteins inside the rod was also evaluated by immersion of specimens in cell culture medium (Dulbecco’s Modified Eagle Medium, DMEM; Gibco, Life Technologies), containing 10% new born calf serum, for 48 h. Protein extraction took place by incubation within a SDS solution (sodium dodecyl sulfate in 10 mM PBS; 2% w/v) for 3 days. Total proteins were colorimetric detected and quantified using the BCA protein assay kit (Thermo Scientific). Proteins reduce Cu^2+^ to Cu^1+^ in alkaline conditions, and two bicinchoninic acid (BCA) molecules chelate the Cu^+1^ ion forming a purple complex that is spectrophotometrically evaluated (λ = 562 nm) with a BIO RAD 680 microplate reader (Microplate ManagerTM Version 5.2 Software). Concentration values (mg·mL^−1^) were obtained from a calibration curve of BSA standard. The average quantities of proteins (n = 6) are reported in μg of proteins per mm^3^ 36. In parallel, nonporous rods made with the same glass that the one used in this study and obtained by Spark Plasma Sintering (SPS) were used as reference to evaluate the protein adsorption at surface level. These nonporous rods were sintered by SPS in vacuum at 750 °C for 3 min at 32 MPa (FCT Systeme GMBH, HPD 25, Germany), following the same procedure that the one previously described by S. López-Esteban *et al*.^37^. Statistical differences between the reference and the investigated rod were assessed (t-Student Test, p ≤ 0.05).

## Additional Information

**How to cite this article**: Moya, J. S. *et al*. Histological response of soda-lime glass-ceramic bactericidal rods implanted in the jaws of beagle dogs. *Sci. Rep.* **6**, 31478; doi: 10.1038/srep31478 (2016).

## Supplementary Material

Supplementary Information
