# Erratum: Histological response of soda-lime glass-ceramic bactericidal rods implanted in the jaws of beagle dogs

**DOI:** 10.1038/srep33425

**Published:** 2016-09-16

**Authors:** José S. Moya, Arturo Martínez, Roberto López-Píriz, Francisco Guitián, Luis A. Díaz, Leticia Esteban-Tejeda, Belén Cabal, Federico Sket, Elisa Fernández-García, Antoni P. Tomsia, Ramón Torrecillas

Scientific Reports 6: Article number: 31478; 10.1038/srep31478 published online: 08 12 2016; updated: 09 16 2016.

This Article contains typographical errors.

In the Results section,

“Very high calcium content appears within the pores (Figs 6a and 2b).”

should read:

“Very high calcium content appears within the pores (Figs 6a2b).”

In the Discussion section,

“Almost all pores were filled with calcium deposits (Figs 6a and 2b), which encourage bone growth^25^.”

should read:

“Almost all pores were filled with calcium deposits (Figs 6a2b), which encourage bone growth^25^.”

