# Supplementary material for: Histological response of soda-lime glass-ceramic bactericidal rods implanted in the jaws of beagle dogs
Source: Sci Rep. 2016 Aug 12;6:31478. doi: 10.1038/srep31478 (PMC4981854; doi:10.1038/srep31478)
Supplement: Supplementary Information [file srep31478-s1.pdf]

Histological response of soda-lime glass-ceramic bactericidal rods implanted in the jaws of beagle dogs

José S. Moya[1], Arturo Martínez[2], Roberto López-Píriz[1], Francisco Guitián[2], Luis A. Díaz[1], Leticia Esteban-Tejeda[3], Belén Cabal[1], Federico Sket[4], Elisa Fernández-García[1], Antoni P. Tomsia[5], Ramón Torrecillas[1*]

[1]Nanomaterials and Nanotechnology Research Center (CINN), CSIC - University of Oviedo (UO), Avda de la Vega 4-6, El Entrego 33940, San Martín del Rey Aurelio, Spain

[2]Galician Institute of Ceramics, Avda Maestro Mateo, 15782 Santiago de Compostela, Spain

[3]Institute of Materials Science of Madrid (ICMM-CSIC), Cantoblanco, 28049, Madrid, Spain

[4] IMDEA Materials Institute, C/ Eric Kandel 2, Getafe, 28906, Madrid, Spain

[5]Materials Sciences Division, Lawrence Berkeley National Laboratory, Berkeley, CA 94720, USA

*Correspondence to: r.torrecillas@cinn.es

Supplementary information

Supplementary Figure Captions

Supplementary Figure 1. Image of glass-ceramic rod showing blood suction by capillary force (photo taken by Roberto López-Píriz during the surgery).

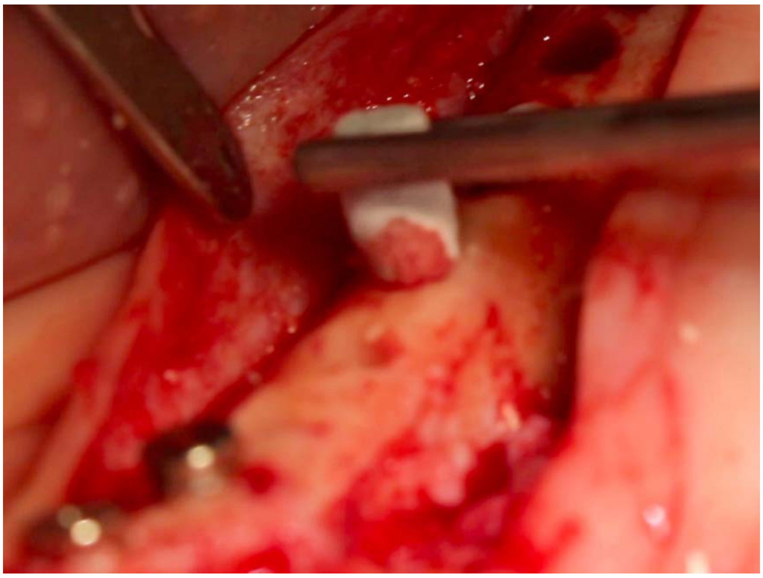

Supplementary Figure 1
